# Electric Field Modulation and Ultrafast Photogenerated Electron-Hole Dynamics in MoSe_2_/WSe_2_ van der Waals Heterostructures

**DOI:** 10.3390/molecules30183840

**Published:** 2025-09-22

**Authors:** Tian-Jun Dai, Zhong-Yuan Fan, Chao-Feng Peng, Xiang Xiao, Yi Zhou, Jian Sun, Zhang-Yu Zhou, Xiang Guo, Xue-Fei Liu, Xiang-Hong Niu

**Affiliations:** 1 School of Electronic Information Engineering, Guiyang University, Guiyang 550005, China; 2 Key Laboratory of Micro-Nano-Electronics of Guizhou Province, College of Big Data and Information Engineering, Guizhou University, Guiyang 550025, China; 3 School of Physics and Electronic Science, Guizhou Normal University, Guiyang 550025, China; 4 School of Science, Nanjing University of Posts and Telecommunications, Nanjing 210023, China

**Keywords:** MoSe_2_/WSe_2_ heterostructures, two-dimensional material, electric field modulation, ultrafast dynamics, nonadiabatic coupling

## Abstract

Understanding the non-equilibrium dynamical processes in two-dimensional (2D) transition metal dichalcogenide (TMDC) heterostructures is essential for elucidating their photoelectric behaviors. In this work, we investigate the electronic structure, electric field modulation, and transient optical performance of the MoSe_2_/WSe_2_ heterostructure using first principles and nonadiabatic molecular dynamics (NAMD) methods. Applying an external electric field effectively modulates the bandgap and band arrangement of MoSe_2_/WSe_2_ heterostructure, along with a transition from indirect to direct bandgap during which the type-II band alignment can be maintained. Specifically, the ultrafast interlayer photogenerated electron transfer time is 72 fs, and the interlayer electron-hole recombination time can be as long as 357 ns, which is longer than that of the intralayer recombination in the constituent monolayers (110 ns for MoSe_2_ and 288 ns for WSe_2_), yielding an ultrahigh charge separation efficiency of up to 99.99%. The significant time difference in the processes of photoinduced charge transfer and recombination can be attributed to the corresponding different nonadiabatic coupling averaged values, mainly determined by the electron–phonon coupling and energy difference. The carrier dynamics mechanism revealed in the MoSe_2_/WSe_2_ heterostructure is conducive to the development of 2D ultrafast optoelectronic devices.

## 1. Introduction

Van der Waals (vdW) heterostructures of two-dimensional (2D) semiconducting transition metal dichalcogenides (TMDCs) materials, featuring strong light-matter interactions and prominent exciton states [1,2,3,4], can be fabricated through chemical vapor deposition [5,6,7] and mechanical transfer [8] approaches experimentally. Optical response properties in such heterostructures are largely determined by excitons (Coulomb attraction between holes and electrons) with large binding energies. Specifically, by engineering TMDC heterostructures with a staggered (type-II) band alignment, the valence band maximum (VBM) and conduction band minimum (CBM) are located in different constituent layers, facilitating the spatial separation of carriers. On irradiation, the photoexcited intralayer excitons in one TMDC layer dissociate into free carriers and are efficiently separated via interfacial charge transfer with ultrafast dynamics, giving rise to bound interlayer excitons. It is noteworthy that, in addition to this charge transfer pathway, direct optical excitation of interlayer excitons has also been demonstrated under resonant conditions in other van der Waals heterostructures [9,10]. These excitons with permanent out-of-plane dipole moments can be modulated through temperature variations [11], twist angle [12], external electric fields [13,14], and pressure engineering [15]. Due to the reduced wavefunctions overlap of electrons and holes resulting from the spatially separated configuration, interlayer excitons exhibit a longer lifetime than intralayer excitons that can extend to even microseconds [16], which holds potential applications for optoelectronic/excitonic devices, quantum circuits, and solar cells [17,18]. Interestingly, the fascinating physics of interfacial charge separation and transfer is not exclusive to vertical van der Waals stacks but is also profoundly important in laterally epitaxially grown heterostructures, where in-plane heterojunctions can efficiently dissociate excitons and generate photocurrent [19].

Transient absorption and photoluminescence (PL) experiments have been extensively employed to examine carrier dynamics (charge transfer and recombination) in TMDCs type-II heterostructures encompassing various combinations. For example, Ji et al. reported an ultrafast interlayer charge transfer of up to ~90 fs in MoS_2_/WS_2_ heterostructure regardless of interlayer coupling strength and twist angles [20]. An ultrafast (~200 fs) temperature-independent charge separation dynamics was demonstrated in a MoSe_2_/WSe_2_ stack, whereas the recombination process displays a strong temperature dependence [21]. Similar ultrafast interlayer exciton formation on a sub-picosecond time scale has also been observed in MoSe_2_/MoS_2_, MoS_2_/MoTe_2_, and WSe_2_/WS_2_ assemblies [22,23,24]. Understanding the transient dynamics and microscopic mechanisms in these heterostructures is of fundamental interest and paramount importance for engineering applications ranging from optoelectronic devices to quantum information. Several potential mechanisms have been proposed to explain the rapid charge transfer processes, involving enhanced coupling of excitation-induced interfacial dipole (albeit with the fact that the directions between the internal electric field and carrier transfer are opposite) [25,26], phonon-mediated intervalley scattering [27,28], and exciton-phonon coupling [29]. However, a comprehensive understanding of the carrier transfer and recombination dynamics within theoretical modeling is still lacking.

In MoSe_2_/WSe_2_ heterobilayers, a prominent representative of TMDCs type-II heterostructures, the moiré-modulated exciton effects render them promising for tunable quantum optics and next-generation opto-excitonic devices [30,31,32,33]. In this paper, we employed density functional theory (DFT) and nonadiabatic molecular dynamics (NAMD) approaches to investigate the electronic structure and optoelectronic characteristics of MoSe_2_/WSe_2_ vdW heterostructures, with particular attention to their photoexcited carrier dynamic. The calculated results show that the MoSe_2_/WSe_2_ heterostructure, with good structural stability, possesses excellent light absorption properties beyond those of the two constituent layers. Moreover, an indirect-to-direct bandgap transition, together with an enhanced band offset, can be realized by varying the electric field from 0.5 to −0.5 V/Å. Bader charge calculations show that 0.0143 e of the charge is migrated from WSe_2_ to MoSe_2_. Importantly, the instantaneous dynamics involving interlayer transfer and interlayer/intralayer recombination are comprehensively examined, and the mechanisms underlying the differences between them are thoroughly revealed, providing a foundation for optoelectronic devices based on MoSe_2_/WSe_2_ heterojunctions.

## 2. Results and Discussion

### 2.1. Electronic Structures and Stability

Before delving into the MoSe_2_/WSe_2_ heterostructure, we initially investigated the geometries and band structures of the constituent monolayers. As shown in Figure S1, both with stable hexagonal structures, the corresponding optimized lattice constants of WSe_2_ and MoSe_2_ are 3.290 Å and 3.294 Å (see Table 1), respectively, aligning with previous experiments [34,35]. A very small lattice mismatch (~0.1%) and the same lattice structure make it feasible to build a MoSe_2_/WSe_2_ heterostructure. A similar shape of band dispersion is observed from the band structures calculated via the PBE and HSE06 approaches (Figure S1b,d). When using the PBE functional, WSe_2_ and MoSe_2_ monolayers exhibit direct bandgaps with values of 1.638 eV and 1.507 eV, respectively, where the conduction band minimums (CBMs) and valence band maximums (VBMs) are located at K point [36,37]. On the contrary, both monolayer WSe_2_ and MoSe_2_ become indirect bandgap systems with larger gaps when calculated with HSE06, in agreement with previous reports [38,39], indicating that our calculations are reliable.

**Figure 1:**
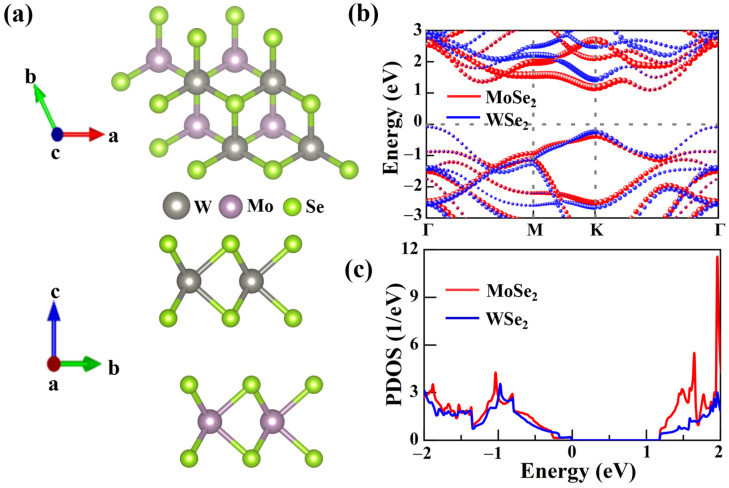
(**a**) Top view and side view of TW configuration for MoSe_2_/WSe_2_ heterostructure. The red and green arrows represent the in-plane lattice directions, while the blue arrow represents the out-of-plane stacking direction. (**b**) The projected band structure of MoSe_2_/WSe_2_ heterostructure using PBE functional. Note that red and blue correspond to MoSe_2_ and WSe_2_, respectively. (**c**) Projected density of states (PDOS) of the MoSe_2_/WSe_2_ heterostructure.

The MoSe_2_/WSe_2_ vdW heterostructure is constructed by employing a 1 × 1 primitive cell of WSe_2_ and a 1 × 1 primitive cell of MoSe_2_ due to their similar lattice constant. Considering that 2D TMDCs heterostructures are usually assembled via chemical vapor deposition and mechanical exfoliation approaches, accompanied by diverse stacking configurations, three typical arrangements, designated as (the W atom of WSe_2_ is located above the Se atoms of MoSe_2_), TSe (the Se atoms of WSe_2_ are located above the Mo atoms of MoSe_2_), and TMo (the Mo and Se atoms of MoSe_2_ are located directly below the W and Se atoms of WSe_2_, respectively) stacks with a 2 × 2 × 1 heterostructure supercell, were considered, as shown in Figure S2. To identify the stability of MoSe_2_/WSe_2_ heterostructures, we calculated their binding energy $E_{b}$ by using the equation: $E_{b}=E_{tot}-E_{MoSe_{2}}-E_{WSe_{2}}$, where $E_{tot}$, $E_{MoSe_{2}}$, and $E_{WSe_{2}}$ represent the total energy of the MoSe_2_/WSe_2_ heterostructure and the two individual monolayers, respectively. The obtained binding energies, interlayer spacings, and bandgaps of these configurations are listed in Table 1. In comparison to the other two stacking configurations, the TW architecture with the smallest interlayer distance (d) has the lowest binding energy, corresponding to the most stable configuration. Note that the smallest interlayer distance indicates the strongest interlayer coupling, facilitating the rapid separation of interlayer charges [40,41]. Consequently, all subsequent investigations were performed on the TW configuration of the MoSe_2_/WSe_2_ heterostructure (Figure 1a).

As shown in Figure 1b, the projected band structure was calculated to evaluate the photoelectric properties of the MoSe_2_/WSe_2_ heterostructure, in which the CBM at the K point originates predominantly from the MoSe_2_ layer, while the VBM at the Γ point stems primarily from the WSe_2_ layer, providing a type II band alignment. This electronic structure facilitates the spatial separation of carriers, with photoexcited electrons and holes preferentially located in opposite layers, leading to the formation of interlayer excitons, reducing the non-radiative recombination, and thus improving the efficiency of optoelectronic devices [20,24,42]. It is found that the MoSe_2_/WSe_2_ heterostructure exhibits a reduced fundamental bandgap (1.183 eV) compared to the isolated layers and is an indirect bandgap, in agreement with other results [21,28]. Although the weak vdW interaction between the two individual monolayers is identified by the electron localization function of the MoSe_2_/WSe_2_ heterostructure (see Figure S3), the projected density of states (PDOS) indicates that WSe_2_ and MoSe_2_ states near the Fermi level are strongly coupled with each other (Figure 1c), facilitating fast electrons and holes movement across the vdW interfaces.

**Figure 2:**
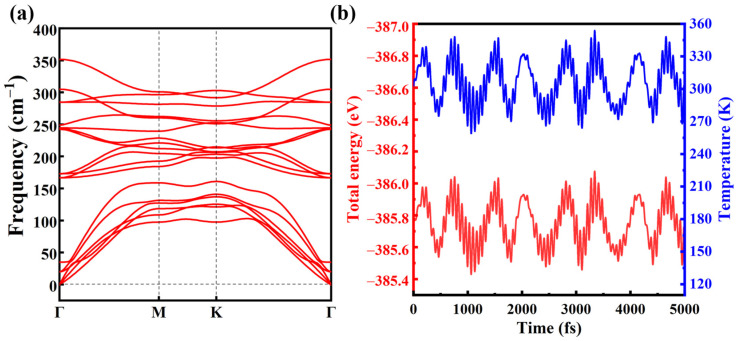
(**a**) The calculated phonon spectra of the MoSe_2_/WSe_2_ heterostructure. (**b**) Fluctuations of the temperature (blue, right axis) and the total energy (red, left axis) during AIMD simulation for the heterostructure at 300 K.

The structural stability of 2D heterostructure materials is vital for the fabrication of optoelectronic devices in experiments. By utilizing a DFPT method, we calculated the phonon dispersion spectra as presented in Figure 2a and Figure S4. No imaginary frequencies are observed in the phonon spectra of the MoSe_2_/WSe_2_ heterostructure and the constituent materials, indicating good lattice dynamical stabilities. Moreover, ab initio molecular dynamics (AIMD) simulations were carried out to validate the thermal stability at 300 K using the canonical ensemble, with a time step of 1 fs sustained to 5000 fs. As exhibited in Figure 2b, only weak fluctuations occur in the total energy of the heterostructure is obtained, and the structural skeleton withstand distortions during the AIMD process, implying that the MoSe_2_/WSe_2_ heterostructure is thermally stable at 300 K. Furthermore, the calculated elastic constants ($C_{ij}$), as summarized in Table S1, satisfy the Born-Huang stability criteria [43]: $C_{11}>\left|C_{12}\right|$, $C_{66}>0$, $C_{22}>0$ and $C_{11}C_{22}>C_{12}^{2}$. Shear modulus, Young’s modulus, and Poisson’s ratio for the heterostructure and the individual layers are also provided in Table S1. These results show that the MoSe_2_/WSe_2_ heterostructure possesses outstanding stability for practical applications.

### 2.2. Effect of External Electric Field

**Figure 3:**
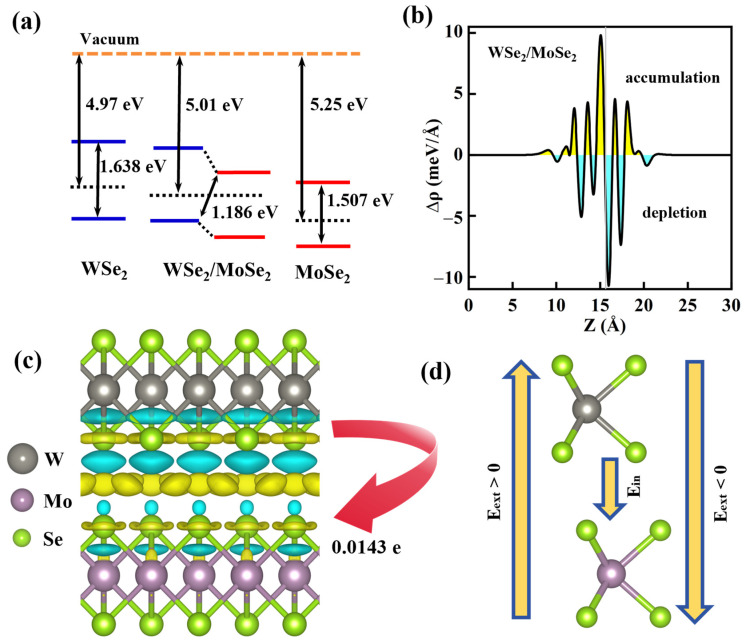
(**a**) Evolution of band edges from the two isolated MoSe_2_ and WSe_2_ monolayers to MoSe_2_/WSe_2_ heterostructure, where the vacuum levels are set to zero. (**b**) The plane-averaged charge density difference along the z-direction for MoSe_2_/WSe_2_ heterostructure, where yellow/cyan indicates the accumulation/depletion of charge. (**c**) Three-dimensional charge density difference of MoSe_2_/WSe_2_ heterostructure; the isosurface values are 0.0002182 e/Å^3^, where yellow/cyan indicates the accumulation/depletion of charge and the red arrow represents the charge transfer direction. (**d**) Schematic illustration of the applied external electric field in the heterostructure.

The evolution of band edges from two individual layers to the MoSe_2_/WSe_2_ heterostructure is presented in Figure 3a, where the work function of MoSe_2_ (5.25 eV) is higher than that of WSe_2_ (4.97 eV) emanating from the calculated electrostatic potential curves along the z-axis (Figure S5), yielding a staggered band arrangement, accompanied by a small conduction band offset ($\Delta E_{c}$) and valence band offset ($\Delta E_{v}$) of 0.491 eV and 0.344 eV, respectively. Accordingly, electrons in WSe_2_ would spontaneously transfer to the MoSe_2_ layer in the combined MoSe_2_/WSe_2_ stack, forming interlayer excitons within permanent electric dipole moments that can be modulated by an external electric field ($E_{ext}$) [16,29]. To further understand the charge redistribution in the MoSe_2_/WSe_2_ assembly, we calculated the charge density difference as follows:

(1) $$∆\rho =\rho_{tot}-\rho_{MoSe_{2}}-\rho_{WSe_{2}}$$

where $\rho_{tot}$, $\rho_{MoSe_{2}}$, and $\rho_{WSe_{2}}$ represent the charge density of the MoSe_2_/WSe_2_ heterostructure, MoSe_2_, and WSe_2_, respectively. As illustrated in Figure 3b, obvious interface charge aggregation occurs in the MoSe_2_/WSe_2_ stacking, with charge accumulation in the MoSe_2_ layer and charge depletion in the WSe_2_ side, which is consistent with the work function analysis. Furthermore, Bader charge analysis indicates that, during the formation of the heterostructure, 0.0143 e charge migrates from the WSe_2_ layer to the MoSe_2_ layer (Figure 3c), which is approximately of the same magnitude as the transferred charge observed in InSe/PtTe_2_ [44], corresponding to a potential difference of 0.09 eV as shown in Figure S6, thereby resulting in an interfacial built-in electric field ($E_{in}$) oriented from WSe_2_ to the MoSe_2_ (see Figure 3d), where the dipole moments of the interlayer excitons point conversely from MoSe_2_ to the WSe_2_ sublayer. Intriguingly, this carrier migration, which can be enhanced by reducing the interlayer spacing and increasing the potential difference between the two isolated layers [45,46], gives rise to a strong exciton absorption peak and light emission quenching as evidenced by PL measurements in vdW heterostructures experimentally [47,48].

**Figure 4:**
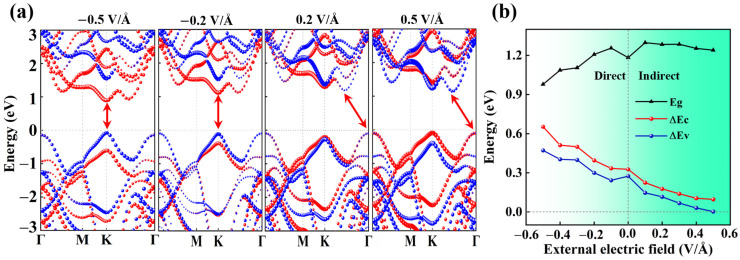
(**a**) Variation of electronic band structures of MoSe_2_/WSe_2_ heterostructure with different electric fields (red on MoSe_2_ and blue on WSe_2_). The red double-headed arrows indicate the changes in the bandgap. (**b**) Dependence of bandgap ($E_{g}$), conduction band offset ($\Delta E_{c}$), and valence band offset ($\Delta E_{v}$) on the applied electric field for MoSe_2_/WSe_2_ heterostructure.

Interestingly, the out-of-plane dipole moments created by interlayer excitons in heterostructures enable electrical modulation of exciton emissions. Variation of the electrostatic doping level allows one to alter the wavefunction overlap of electrons and holes, impacting the interlayer excitons’ lifetime [16], thereby regulating the optical properties. We investigated the electrical modulation by applying an external electric field perpendicularly to the MoSe_2_/WSe_2_ heterostructure, where the field pointing from MoSe_2_ to WSe_2_ ($E_{ext}$ > 0) corresponds to the positive direction, counteracting the built-in electric field, as illustrated in Figure 3d. Predictably, the applied electric field, varying from −0.5 to 0.5 V/Å with a step of 0.1 V/Å, has a significant influence on the band structures of the MoSe_2_/WSe_2_ heterostructure, as displayed in Figure 4a and Figure S7. It is found that the CBM and VBM positions of MoSe_2_ shift towards the higher energy regions as the $E_{ext}$ increases, while those of WSe_2_ change in the opposite direction. The type-II band arrangement can be maintained during changes in $E_{ext}$, where the CBM and VBM of the heterostructure are, respectively, contributed by MoSe_2_ and WSe_2_ until the $E_{ext}$ exceeds 0.3 V/Å, at which point their roles are reversed. Correspondingly, the dependence of band offsets ($∆E_{c}$ and $∆E_{v}$) and bandgap ($E_{g}$) for the MoSe_2_/WSe_2_ heterostructure on $E_{ext}$ is plotted in Figure 4b. Evidently, when a positive $E_{ext}$ is provided, the $E_{g}$ of the heterostructure undergoes only slight changes, while it drops monotonically with decreasing $E_{ext}$ under a negative electric field and even decreases to 0.978 eV at the −0.5 V/Å point, leading to a red-shift of the optical absorption edge [44]. Specifically, an electric field applied parallel to the dipole moments can reduce the interlayer exciton recombination rate, resulting in the observed red-shift in the PL energy of interlayer excitons [14,16]. In addition, the observation of an indirect to direct bandgap transition under $E_{ext}$ varying from 0.5 to −0.5 V/Å indicates the presence of strong interlayer coupling [49], along with a greatly enhanced $\Delta E_{c}/\Delta E_{v}$ which enables the MoSe_2_/WSe_2_ heterostructure to efficiently separate the photogenerated carriers, thereby enabling high-performance optoelectronic devices.

### 2.3. Optical Absorption

**Figure 5:**
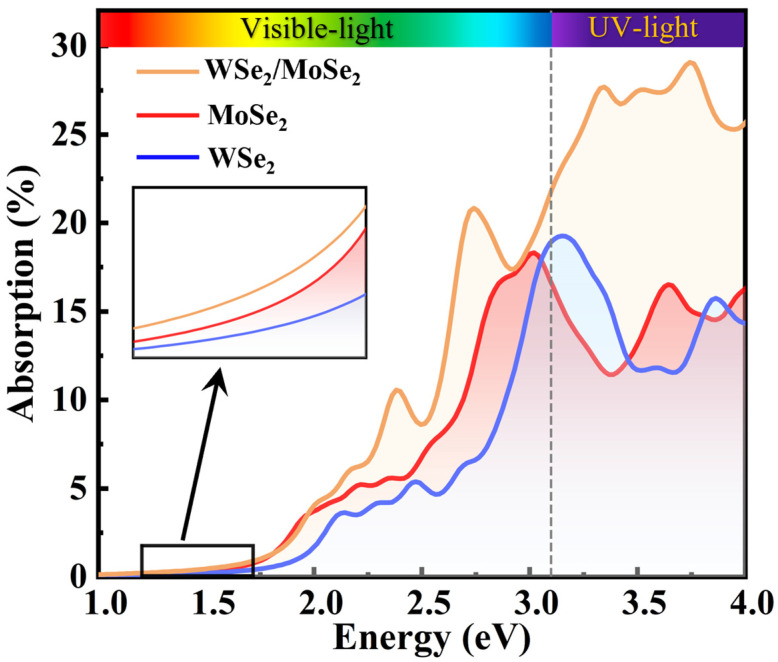
Optical absorption spectra of MoSe_2_/WSe_2_ heterostructure and the two sublayers calculated by using HSE06 functional. The different range of the spectra is marked with a dashed line.

For 2D thin films with atomic layer thickness, the light absorption properties are crucial for their optoelectronic devices. The absorption coefficient, $\alpha \left(\omega \right)$, is employed to evaluate the optical properties, which can be calculated using the following equation [44]:

(2) $$\alpha \left(\omega \right)=\sqrt{2}\omega {\left[\sqrt{\varepsilon_{1}^{2}\left(\omega \right)+\varepsilon_{2}^{2}(\omega )}-\varepsilon_{1}\left(\omega \right)\right]}^{\frac{1}{2}}$$

where ω, $\varepsilon_{1}\left(\omega \right)$, and $\varepsilon_{2}\left(\omega \right)$ denote the frequency of light and the real part and imaginary part of the dielectric function, respectively (see Note S1 in Supplementary Materials). Accordingly, by using the HSE06 method, the optical absorption spectra of the individual MoSe_2_ layer and WSe_2_ layer, as well as the MoSe_2_/WSe_2_ heterostructure, were calculated and are shown in Figure 5. Distinguishable absorption peaks can be observed in all spectra, indicating strong light-matter interactions [50], where the maximum absorption peaks of MoSe_2_ and WSe_2_ monolayers appear at 3.002 eV and 3.15 eV, with corresponding absorption efficiencies of 18.26% and 19.27%, respectively. In the range of 1–3 eV, MoSe_2_ exhibits higher absorption efficiency than that of WSe_2_. Additionally, after forming the MoSe_2_/WSe_2_ heterostructure, the maximum absorption peak shifts to 3.76 eV with a corresponding absorption efficiency of up to 29.0%, exhibiting approximately two times higher light absorption than that of the two individual monolayers in the ultraviolet (UV) light region. Similar phenomena have also been observed in other 2D heterostructures, such as WS_2_/MoSe_2_ [45] and WSe_2_/MoSi_2_N_4_ [51]. Moreover, a broadened visible light absorption range is obtained for the MoSe_2_/WSe_2_ heterostructure, which can be attributed to the presence of interlayer excitons, which lead to relatively small exciton binding energy [44,50]. These results show that the MoSe_2_/WSe_2_ heterostructure has excellent absorption properties and is promising for high-performance optoelectronic devices.

### 2.4. Photoexcited Carrier Dynamics

**Figure 6:**
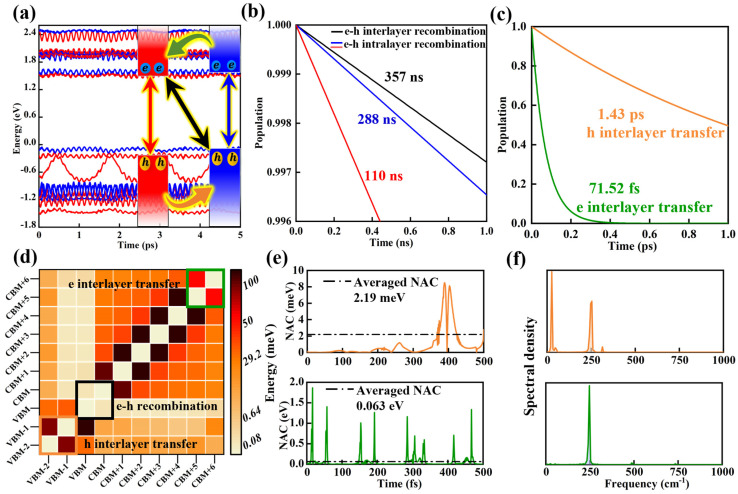
(**a**) Time evolution of the energy states of MoSe_2_/WSe_2_ heterostructure around Fermi level. The Fermi level is set to zero. The blue and red lines represent the energy states of WSe_2_ and MoSe_2_, respectively. The interlayer (intralayer) recombination and transfer processes of photoexcited carriers are indicated by black (blue and red), orange (holes), and green (electrons) arrows. (**b**) The recombination and (**c**) transfer of the photoexcited holes and electrons. For the intralayer recombination, the red and blue lines correspond to MoSe_2_ and WSe_2_ respectively. (**d**) Averaged absolute values of NAC between different electronic states. (**e**) The time dependence of NAC for the photoexcited holes (orange line) and electrons (green line) interlayer transfer. The averaged value of NAC is marked by the black dashed line. (**f**) Fourier transforms of autocorrelation functions for the fluctuations of the energy between electronic states, orange for photogenerated hole interlayer transfer and green for photogenerated electron interlayer transfer.

Before performing the ultrafast excited-state dynamic simulation, we obtained the band structure of the MoSe_2_/WSe_2_ heterostructure based on a 3×3×1 supercell (see Figure S8a in the Supplementary Materials). In this structure, the MoSe_2_ VBM (WSe_2_ CBM) couples and mixes with multiple WSe_2_ (MoSe_2_) states (see Figure S8b), leading to the formation of delocalized initial states between the two layers for carrier transfer processes, which facilitates ultrafast charge transfer across the vdW heterostructure [21]. To visualize the interlayer transfer and recombination process of photoexcited electrons and holes (PEHs), as shown in Figure 6a, we initiated the NAMD calculations with photogenerated electrons (holes) localized on the CBM (VBM) energy states of MoSe_2_/WSe_2_ heterostructure. Obviously, the photoexcited carrier transfer happens in the two different layers by an ultrafast pattern, albeit with the weakly coupled vdW heterointerfaces. For the recombination process, as exhibited in Figure 6b, the timescale of the interlayer recombination can be approximately estimated to be about 357 ns by employing the exponential function $P\left(t\right)=exp(-t/\tau )$ [52], which is longer than that of the intralayer recombination (110 ns for MoSe_2_ and 288 ns for WSe_2_), indicating a larger interlayer exciton lifetime compared to the intralayer excitons, and, thus, the interlayer excitons can diffuse over micrometer-length scales [18,53]. Moreover, an ultrafast interlayer transfer process is observed at the MoSe_2_/WSe_2_ type-II heterostructure interface. Within 1.43 ps, ultrafast interlayer hole transfer occurs from the MoSe_2_ layer to the WSe_2_ layer, whereas ultrafast photogenerated electron transfer takes place in the opposite direction within 71.52 fs (see Figure 6c), which is much faster than that of the MoS_2_/WSe_2_ heterostructure (470 fs) [50], the Janus-MoSSe/WS_2_ heterostructure (ranging from 286 fs to 1.03 ps), and MoS_2_/MoSe_2_ heterostructure (sub-picosecond) [54,55]. Additionally, by utilizing a formula, $1-\tau_{tr}/\tau_{re}$ [56], where $\tau_{re}$ and $\tau_{tr}$ denote recombination time and the transfer time of the PEHs, respectively, the internal quantum efficiency is calculated to be as high as 99.99%, indicating an ultrahigh charge separation efficiency.

For the recombination/transfer between different electronic states, the hopping probability of PEHs is mainly determined by the nonadiabatic coupling (NAC) elements, which can be described as follows [52]:

(3) $$d_{jk}=\langle \varphi_{j}\left|\frac{\partial }{\partial t}\right|\varphi_{k}\rangle =\frac{\langle \varphi_{j}\left|\nabla_{R}H\right|\varphi_{k}\rangle }{\varepsilon_{k}-\varepsilon_{j}}\dot{R}$$

where H is the Kohn–Sham Hamiltonian; $\dot{R}$ is the velocity of the nuclei; and $\varphi_{k}$, $\varphi_{j}$, $\varepsilon_{k}$, and $\varepsilon_{j}$ are the wave functions and corresponding eigenvalues for the k and j electronic states. Obviously, the NAC is negatively correlated with the energy difference ($\varepsilon_{k}-\varepsilon_{j}$), but positively correlated with the electron–phonon coupling term $\langle \varphi_{j}\left|\nabla_{R}H\right|\varphi_{k}\rangle$ and $\dot{R}$ (depends on temperature). Considering that the temperature is identical for the PEHs, the first two factors will play an important role in regulating the NAC. Figure 6d shows the averaged absolute values of the NAC between different electronic states covering the band edges of each sublayer. The NAC between neighboring states is stronger than that between separated states for interlayer transfer, while the opposite is true for recombination, demonstrating that the interlayer transfer or recombination process primarily occurs between the neighboring states, marked by three block diagrams. For photogenerated electron and hole transfer processes, as shown in Figure 6e, the averaged values of NAC are 63 meV and 2.19 meV, respectively. This is significantly larger than that of interlayer recombination (0.054 meV, see Figure S9a in the Supplementary Materials), resulting in a huge time difference between interlayer transfer (ultrafast) and exciton lifetime (long).

To further reveal the intrinsic influencing factors of NAC, we visualized the corresponding phonon modes coupling with electronic states by using Fourier transforms (FT) of the autocorrelation functions (see Figure 6f and Figure S9b). Clearly, a dominant phonon mode with a frequency around 250 cm^−1^ (out-of-plane vibration mode) for photogenerated electron transfer is observed, which is more robust than that of hole transfer and recombination processes. It is found that the hole transfer and recombination processes are governed mainly by a strong low-frequency phonon pattern near 26 cm^−1^ (shear mode), yielding a longer time scale than that of interlayer electron transfer. Interestingly, the main phonon modes, whether for PEHs transfer or recombination, are concentrated in the low frequency modes below 252 cm^−1^, which mainly stem from the strong interlayer interaction. Moreover, the NAC elements are also associated with the energy difference between acceptor and donor states. The energy difference for photogenerated electron interlayer transfer in the MoSe_2_/WSe_2_ heterostructure is notably smaller than that of hole interlayer transfer and PEHs interlayer recombination (see Figure 6a), providing another reason for the fastest rate of interlayer electron transfer.

## 3. Computational Methods

All computations were performed by using the density functional theory (DFT), which was realized in the Vienna Ab-Initio Simulation Package (VASP) code. The electron–ion interactions were characterized by using the projected augmented wave pseudopotential (PAW) [57,58]. The exchange–correlation interaction was calculated using the Perdew–Burke–Ernzerhofer (PBE) functional with generalized gradient approximation (GGA) [59] for geometry optimization, electronic structure, and structural stability. The kinetic energy cutoff was set to 600 eV. The convergence criteria of the total energy and the force on each atom were set to 10^−7^ eV and 10^−2^ eV, respectively. In order to optimize the structure fully, we adopted the two-step optimization method consisting of first low-precision and then high-precision calculations. We used different k-point grids to sample the Brillouin zones for the monolayers (18 × 18 × 1) and heterostructures (15 × 15 × 1). To prevent interactions between periodic sublayers, a vacuum thickness of 20 Å was utilized along the z-direction. To obtain accurate optical properties, the hybrid Heyd–Scuseria–Ernzerhof (HSE06) hybrid functional was employed [60]. Furthermore, the phonopy code was utilized to compute the phonon dispersion curves, which were calculated by implementing the density functional perturbation theory (DFPT) method within a 3 × 3 × 1 supercell, and the energy convergence criterion was set to be 10^−8^ eV [61]. To describe the vdW weak interaction in the 2D materials [62], the semiempirical dispersion-corrected DFT-D3 method proposed by Grimme was employed.

The dynamics of photogenerated carriers were obtained by the ab initio nonadiabatic molecular dynamics (NAMD) within time-domain DFT using the Hefei-NAMD code [63]. Supercells consisting of 3 × 3 × 1 MoSe_2_/WSe_2_ unit cells were used to simulate the dynamic process. For the optimized heterostructure, we heated it to 300 K for 3 ps based on the repeated velocity rescaling approach and obtained a 5 ps microcanonical trajectory with a time step of 1 fs via ab initio molecular dynamics (AIMD). The corresponding wave function was generated as well. The dynamic evolution processes of photogenerated carriers were acquired by averaging over 100 different initial configurations and sampling 2 × 10^4^ trajectories for each MD trajectory. The quantum-classical decoherence-induced surface hopping algorithm (DISH) was employed to provide a probability for hopping between interacting states based on the evolution of the adiabatic basis coefficients [64].

## 4. Conclusions

In summary, through DFT and NAMD computations, we have systematically explored the electronic band structures, electric field regulation, light absorption, and photoinduced carrier dynamics of the MoSe_2_/WSe_2_ vdW heterostructure. We demonstrated efficient tuning of the band structures by applying an external electric field and observed a clear evolution of CBM and VBM positions accompanied by a field-induced bandgap transition while simultaneously maintaining the type-II band alignment, which facilitates spatial separation of PEHs. Significantly, the MoSe_2_/WSe_2_ heterostructure exhibited an absorption efficiency of up to 29.0%, which is much higher than that of the constituent monolayers, indicating excellent optical absorption properties. In addition, it was determined that photogenerated electron transfer from WSe_2_ to MoSe_2_ occurred within 71.52 fs, which is faster than that of the interlayer hole transfer (1.43 ps), and a large interlayer photogenerated carrier lifetime of 357 ns was obtained. The significant differences in the transfer and recombination rates of photogenerated carriers resulted from the difference in the averaged NAC values, involving electron–phonon coupling and the energy difference terms. Our findings provide vital insights into the design of novel ultrafast optoelectronic devices for MoSe_2_/WSe_2_ heterostructures.

## Figures and Tables

**Table 1:** The lattice constants (a/b), interlayer distance (d), binding energy ($E_{b}$), and band gap calculated by PBE ($E_{g}^{PBE}$) and HSE06 ($E_{g}^{HSE}$) methods for MoSe_2_/WSe_2_ heterostructure and the two constituent monolayers.

	a/b (Å)	d (Å)	$E_{b}$ (eV)	$E_{g}^{PBE}$ (eV)	$E_{g}^{HSE}$ (eV)
WSe_2_	3.290	—	—	1.638	2.023
MoSe_2_	3.294	—	—	1.507	2.122
TMo	3.278	3.654	−0.211	1.293	1.705
TSe	3.281	3.022	−0.316	1.139	1.602
TW	3.281	3.013	−0.317	1.183	1.752

## Data Availability

The original contributions presented in this study are included in the article/Supplementary Material. Further inquiries can be directed to the corresponding author(s).

## Supplementary Materials

The following supporting information can be downloaded at: https://www.mdpi.com/article/10.3390/molecules30183840/s1. Figure S1. Top and side views of the (a) MoSe_2_ and (c) WSe_2_ primitive cell. Band structures of (b) isolated MoSe_2_ and (d) WSe_2_ monolayer calculated by using both PBE and HSE06 approaches; Figure S2. The top and side views of MoSe_2_/WSe_2_ heterostructure with different stacking orders: (a) TMo, the Mo and Se atoms of MoSe_2_ are located directly below the W and Se atoms of WSe_2_, respectively, (b) TSe, the Se atoms of WSe_2_ is located top the Mo atoms of MoSe_2_, and (c) TW, the W atom of WSe_2_ is located top the Se atoms of MoSe_2_; Figure S3. Electron localization function for MoSe_2_/WSe_2_ heterostructure; Figure S4. Phonon spectra of the MoSe_2_ and WSe_2_ monolayers; Figure S5. The electrostatic potentials of the monolayer (a) WSe_2_ and (b) MoSe_2_ in the z-direction; Figure S6. The planar-averaged electrostatic potential curve of the MoSe_2_/WSe_2_ heterostructure along the z axis; Figure S7. Projected band structures of MoSe_2_/WSe_2_ heterostructure with varied external electric fields. The blue and red dotted lines denote the contribution of WSe_2_ and MoSe_2_, respectively; Figure S8. (a) Top and side views of the 3 × 3 × 1 MoSe_2_/WSe_2_ heterostructure supercell, and (b) the corresponding band structure. The blue and red dotted lines denote the contribution of WSe_2_ and MoSe_2_, respectively; Figure S9. (a) The time-dependent evolution of NAC between electronic states dominating interlayer recombination in the MoSe_2_/WSe_2_ heterostructure. The black dash line represents the averaged value of NAC. (b) Fourier transforms of autocorrelation functions for the fluctuations of the energy between electronic states involving the photogenerated carriers interlayer recombination; Table S1. Elastic constants *C**i**j*, Shear Modulus (G), Young’s modulus (E) and Poisson’s ratio (v) of MoSe_2_/WSe_2_ heterojuntion and the two isolated layers [65].
