# Supplementary material for: Electric Field Modulation and Ultrafast Photogenerated Electron-Hole Dynamics in MoSe2/WSe2 van der Waals Heterostructures
Source: Molecules. 2025 Sep 22;30(18):3840. doi: 10.3390/molecules30183840 (PMC12472952; doi:10.3390/molecules30183840)
Supplement: Supplementary file 1 [file molecules-30-03840-s001.zip › molecules-3862243-supplementary.pdf]

Supporting information

Electric Field Modulation and Ultrafast Photogenerated Electron-Hole Dynamics in $MoSe_2/WSe_2$ van der Waals Heterostructures

Tian-Jun Dai[a*], Zhong-Yuan Fan[a], Chao-Feng Peng[b], Xiang Xiao[a], Yi Zhou[a], Jian Sun[a], Zhang-Yu Zhou[a], Xiang Guo[b], Xue-Fei Liu[c*], Xiang-Hong Niu[d*]

[a]School of Electronic Information Engineering, Guiyang University, Guiyang 550005, China

[b]Key Laboratory of Micro-Nano-Electronics of Guizhou Province, College of Big Data and Information Engineering, Guizhou University, Guiyang, 550025, China

[c]School of physics and electronic science, Guizhou Normal University, Guiyang, 550025, China

[d]School of Science, Nanjing University of Posts and Telecommunications, Nanjing 210023, China

*Corresponding authors.

Tian-Jun Dai (*E-mail:*daitianjun@gyu.edu.cn)

Xue-Fei Liu (*E-mail:*201307129@gznu.edu.cn)

Xiang-Hong Niu (*E-mail:*xhniu@njupt.edu.cn)

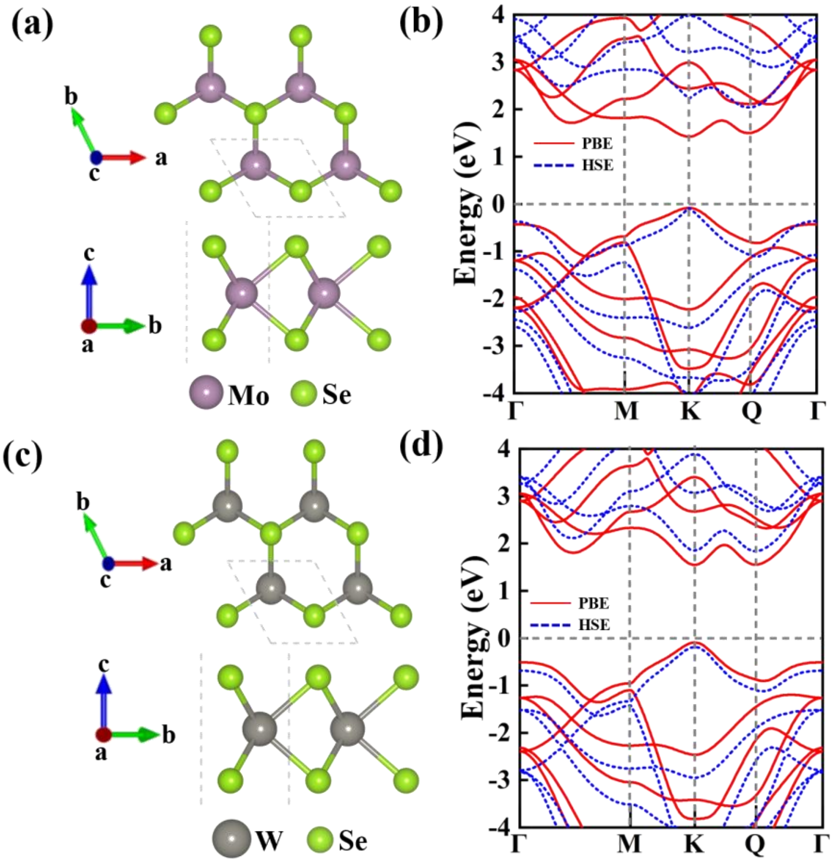

Fig. S1. Top and side views of the (a) $MoSe_2$ and (c) WSe_2 primitive cell. Band structures of (b) isolated $MoSe_2$ and (d) WSe_2 monolayer calculated by using both PBE and HSE06 approaches.

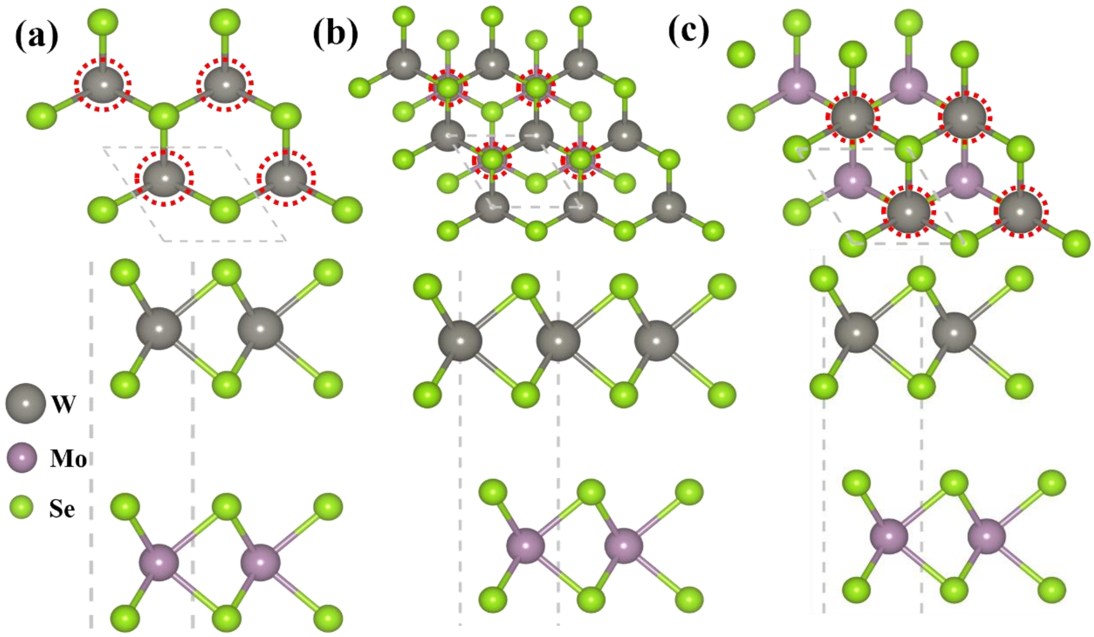

Fig. S2. The top and side views of $MoSe_2/WSe_2$ heterostructure with different stacking orders: (a) TMo, the Mo and Se atoms of $MoSe_2$ are located directly below the W and Se atoms of WSe_2, respectively, (b) TSe, the Se atoms of WSe_2 is located top the Mo atoms of $MoSe_2$, and (c) TW, the W atom of WSe_2 is located top the Se atoms of $MoSe_2$.

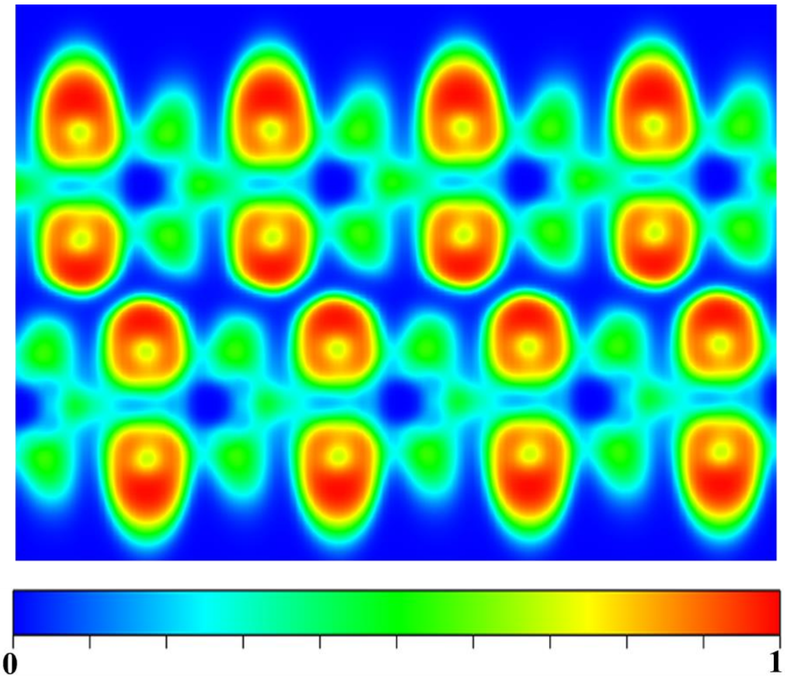

Fig. S3. Electron localization function for $MoSe_2/WSe_2$ heterostructure.

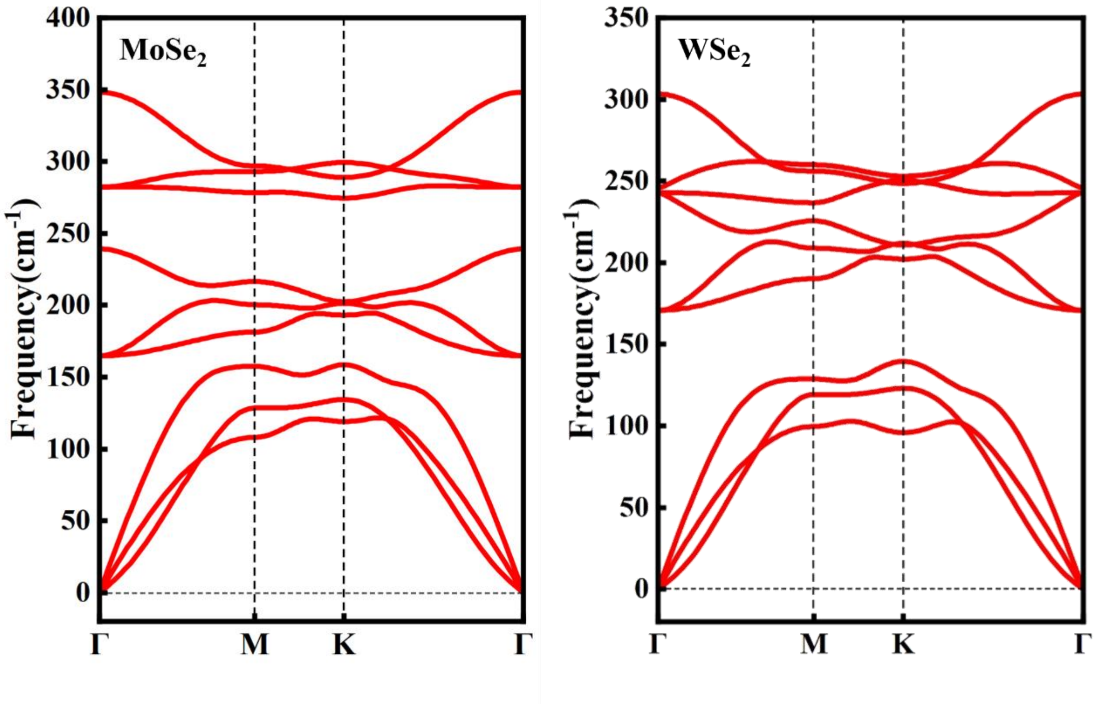

Fig. S4. Phonon spectra of the $MoSe_2$ and WSe_2 monolayers.

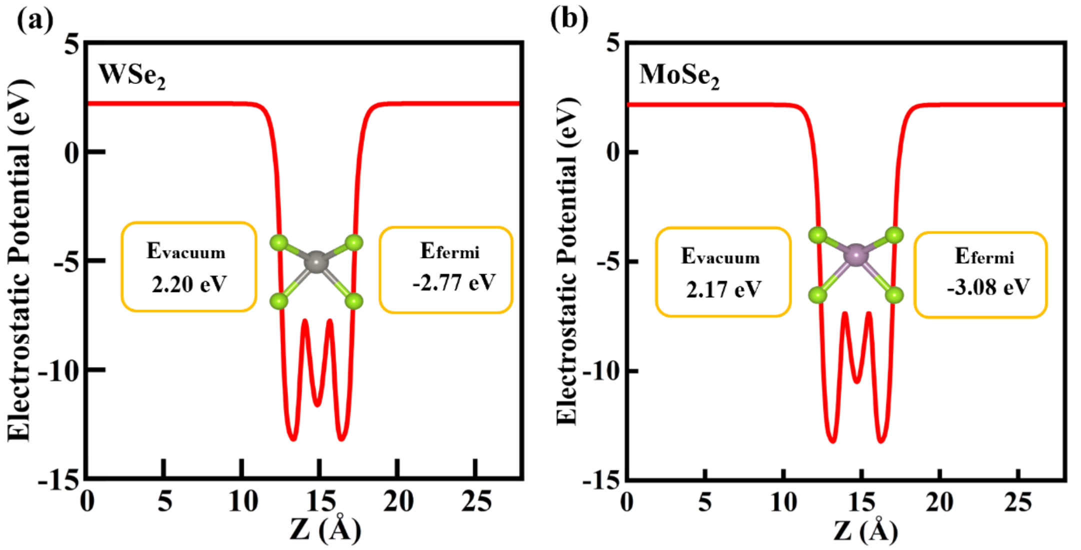

Fig. S5. The electrostatic potentials of the monolayer (a) WSe_2 and (b) $MoSe_2$ in the z-direction.

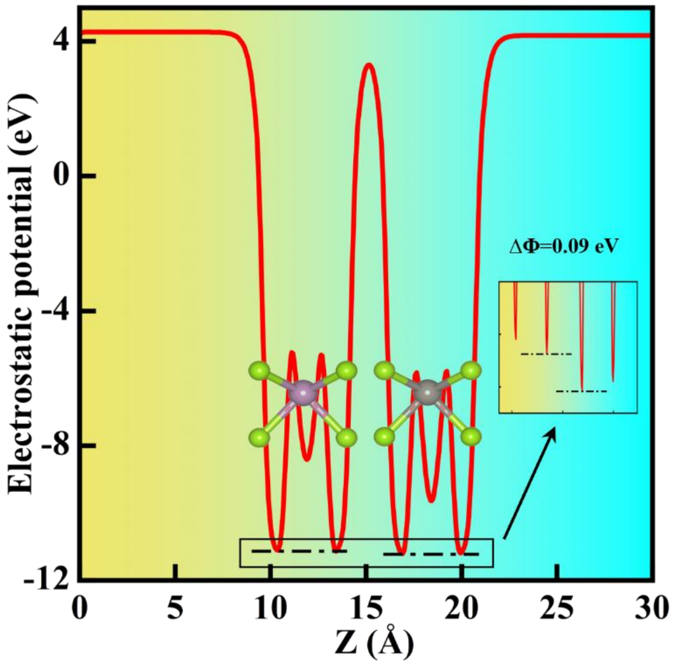

Fig. S6. The planar-averaged electrostatic potential curve of the $MoSe_2/WSe_2$ heterostructure along the z axis.

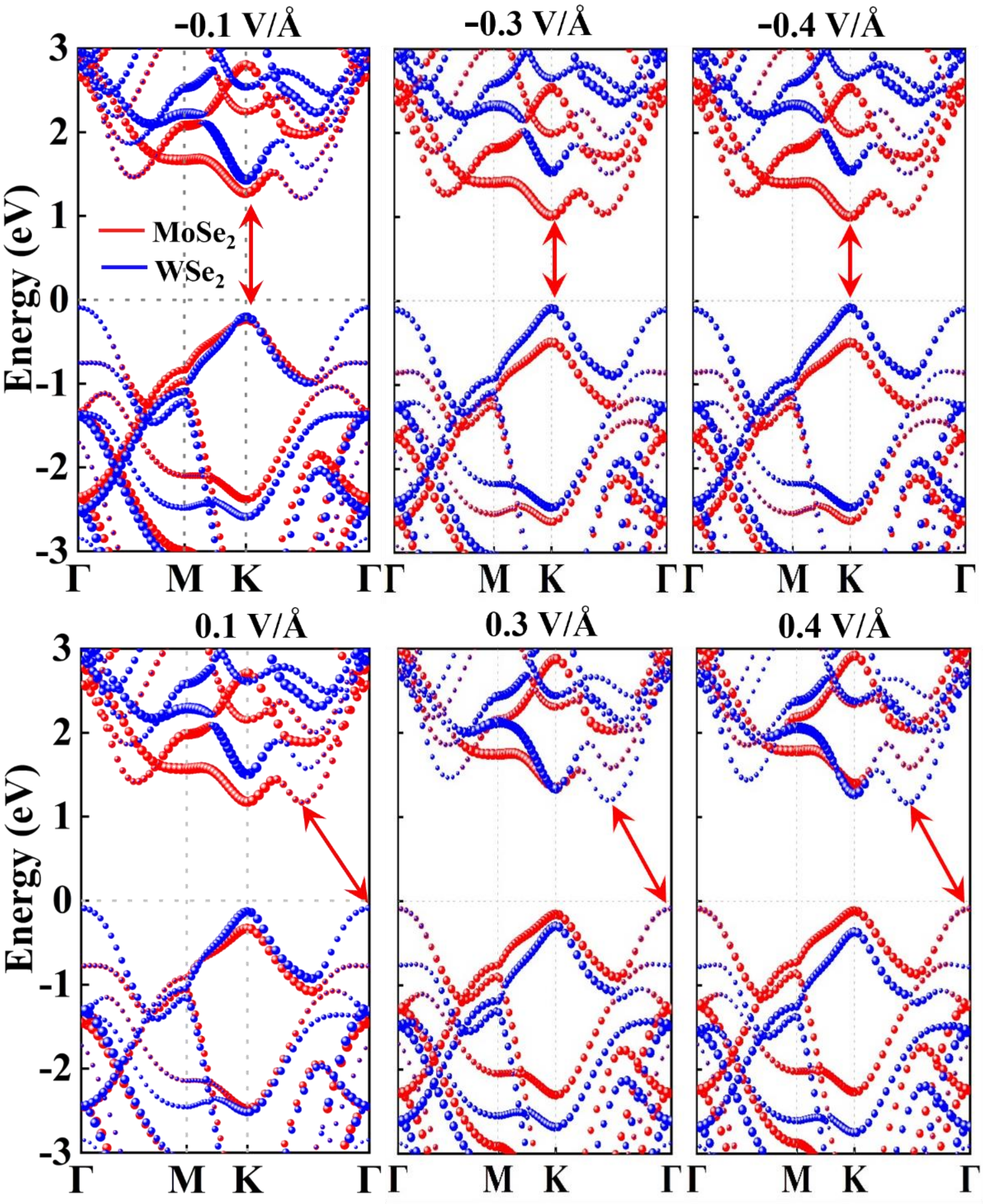

Fig. S7. Projected band structures of $MoSe_2/WSe_2$ heterostructure with varied external electric fields. The blue and red dotted lines denote the contribution of WSe_2 and $MoSe_2$, respectively.

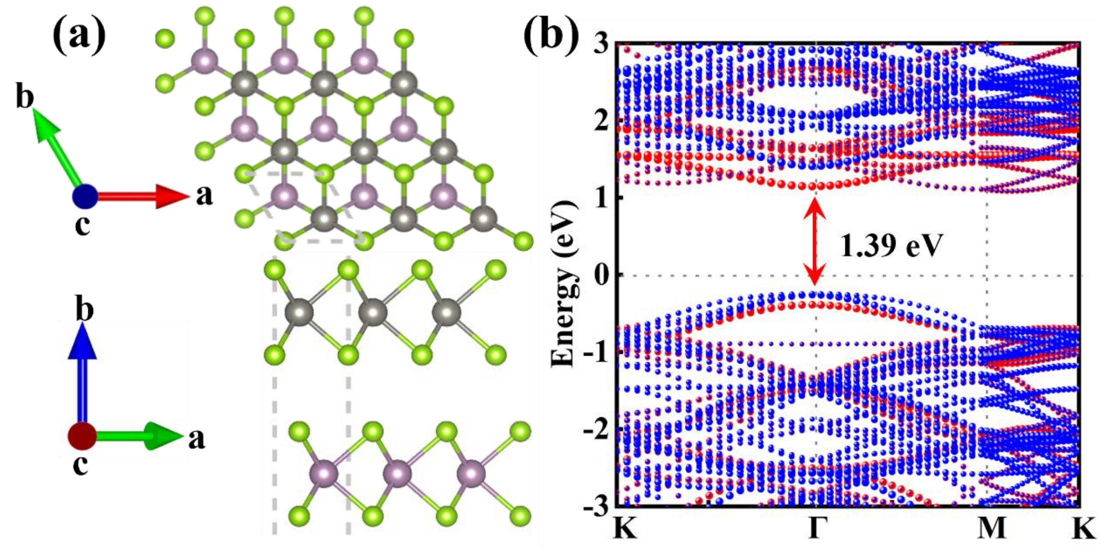

Fig. S8. (a) Top and side views of the $3 \times 3 \times 1$ $MoSe_2/WSe_2$ heterostructure supercell, and (b) the corresponding band structure. The blue and red dotted lines denote the contribution of WSe_2 and $MoSe_2$, respectively.

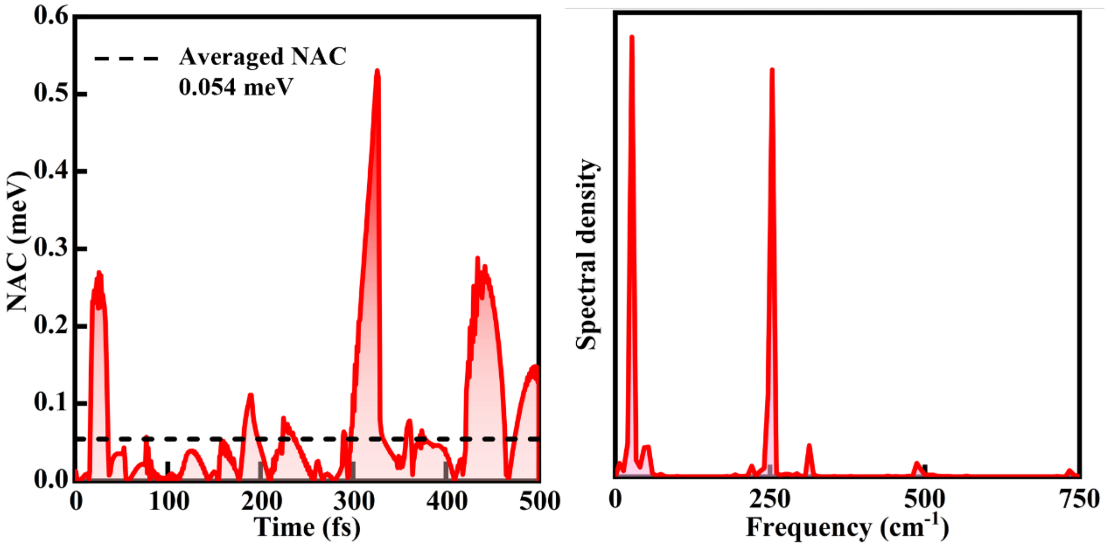

Fig. S9. (a) The time-dependent evolution of NAC between electronic states dominating interlayer recombination in the MoSe2/WSe2 heterostructure. The black dash line represents the averaged value of NAC. (b) Fourier transforms of autocorrelation functions for the fluctuations of the energy between electronic states involving the photogenerated carriers interlayer recombination.

Table S1. Elastic constants C_{ij}, Shear Modulus (G), Young's modulus (E) and Poisson's ratio (v) of $MoSe_2/WSe_2$ heterojuntion and the two isolated layers.

System	C_{11} (N/m)	C_{12} (N/m)	C_{66} (N/m)	E (N/m)	G (N/m)	v
WSe_2	162.301	43.297	59.502	150.750	59.502	0.267
$MoSe_2$	147.774	43.176	52.299	135.159	52.299	0.292
TW	311.419	87.560	111.930	286.800	111.930	0.281

Note S1:

The frequency-dependent complex dielectric function $\varepsilon(\omega) = \varepsilon_1(\omega) + i\varepsilon_2(\omega)$ can be used to calculate optical properties. The momentum matrix elements between occupied and unoccupied states can be leveraged to identify the imaginary part $\varepsilon_2(\omega)$, whereas the real part of the dielectric function $\varepsilon_1(\omega)$ can be determined from the Kramers-Kronig expressions[1]:

$$\varepsilon_2(\omega) = \frac{2e^2\pi}{\Omega\epsilon_0}\sum_{k,v,c}\delta(E_k^c - E_k^v - E)|\langle\psi_k^c|\hat{u} \times \hat{r}\,|\psi_k^v\rangle|^2 \quad (1)$$

$$\varepsilon_1(\omega) = 1 + \left(\frac{2}{\pi}\right)\int_0^{\infty}\frac{\omega'^2\varepsilon_2(\omega')}{\omega'^2-\omega^2}d\omega' \quad (2)$$

Where ω, $\hat{u}$, e, ψ_k^c and ψ_k^v are the frequency of light, vectors that characterize the polarization of the incident electric field, electronic charge, and the wave functions of the conduction band and valence band, respectively.

Reference

1. Gajdoš, M.; Hummer, K.; Kresse, G.; Furthmüller, J.; Bechstedt, F. Linear Optical Properties in the Projector-Augmented Wave Methodology. *Phys. Rev. B* **2006** 73, 045112.
